# Reviewed article: Pio IDSL, Nunes DM, Barros JF, Silva FS. Development and validation of an instrument for tracking and analyzing recovery from symptoms associated with COVID-19. Epidemiol Serv Saude. 2025:34;e20240220.

**DOI:** 10.1590/S2237-96222025v34e20240220.a

**Published:** 2025-08-04

**Authors:** Cristiano Bertolossi Marta

**Affiliations:** Universidade do Estado do Rio de Janeiro, Fundamentos de Enfermagem

**Table:** 

Rating	Excellent	Good	Average	Below average	Poor
Interest	✓				
Quality	✓				
Originality	✓				
Overall	✓				

**Table:** 

Questionnaire	Yes	No	Not applicable
Does the manuscript contain new and significant information to justify publication?	✓		
Does the Abstract (Summary) clearly and accurately describe the content of the article?	✓		
Is the problem significant and concisely stated?	✓		
Are the methods described comprehensively?	✓		
Are the interpretations and conclusions justified by the results?	✓		
Is adequate reference made to other work in the field?	✓		

## Manuscript Structure

Length of article is: Adequate

Number of tables is: Adequate

Number of figures is: Adequate

Please state any conflict(s) of interest that you have in relation to the review of this paper (state “none” if this is not applicable): None

## Comments

Prezados,

A pesquisa possui relevância para a área da saúde e principalmente para o indivíduo que usa o sistema. Ela traz evidências que permitirão a tomada de cisão por parte dos gestores sobre a prática de seus pares.

Parabéns!! 

